# Dr. Kinglake, on Mr. Mace's Case of Gout

**Published:** 1805-04-01

**Authors:** Robert Kinglake

**Affiliations:** Taunton


### *To the Editors of the Medical and Physical Journal.*

GENTLEMEN,

THE narrative of Mr. Mace's case, detailed in the last number of your Journal, by Messrs. Scott and Taynton, is replete with valuable information on the refrigerant treatment of gout. In this case, irregular symptoms arose in the practice highly worthy of comment.

The diversity of the human temperament is so vastly extensive, with respect to the motive powers of life, that it is but natural to expect in the same general disease much occasional variation from the common standard, both in the mode of the affection, and in the influence of the means which might be instituted for its cure. A marked peculiarity presents in the constitutional manner in which gouty inflammation had uniformly affected the patient alluded to. It is asserted, that in all former instances or the disease, and when the cooling treatment was not employed, the same chain of morbid irritation prevailed. This diseased state of sympathetic action seems, by long continuance, to have acquired a fixed establishment, not readily to be subdued by the prompt removal of the local excitement, which appears to have produced it. No uneasiness is stated to have disturbed any of the visceral parts previously to the appearance of inflammatory gout on the joints, either in the recent instances under consideration, or on any former occasion; it is therefore fair to infer, that this affection must have been solely symptomatic of the local pain; nor does the circumstance of visceral disease remaining after the removal of the local pain, at all militate against this conclusion.

Morbid actions, however generated, or however linked by sympathetic connection, neither arise nor cease at the same time throughout their whole extent, but begin, increase, diminish, and terminate by portions. Thus, a locked-jaw, resulting from a punctured ligament or tendon, does not cease with the discontinuance of the local torture which induced it. The wound which caused the spasmodic affection may (as the source or first link in the chain of morbid excitement) be healed, yet the diseased state of motive power, pervading the intermediate parts from the seat of grievance to the muscular structure of the jaw, might still remain, and exert with undiminished force its noxious influence.

If habitual gout in any joint should engender diseased sympathy with the visceral organs, the menacing effect of the association will be liable to be shewn in symptoms of reciprocal disorder. This consideration furnishes the most cogent argument for promptly subduing the local disease, lest its protracted existence should open and eventually establish a hazardous state of morbid intercourse with parts more immediately essential to vitality.

The train of morbid evil that constitutes sympathetic ailment in the animal economy is not curable, or even safely transferable, by encouraging and sustaining it on any portion of the disordered parts. The intimate connection, the continuity indeed subsisting between all the parts which may be associated in sympathetic disease, must necessarily render every point of its extent subject to be influenced by an impression made on any portion of it; hence the dangerous tendency of fostering one disease, with a view to the removal of another, and the culpable neglect neglect of indulging inflammatory excitement, or gout, on the joints, in the delusive hope of either expelling or obviating visceral disease by its *stationed* agency.

Though no difficulty occurs in assigning a rational cause for the peculiar organic affection, which attended Mr. Mace's case, during both its natural progress and the refrigerant treatment, yet it was highly important to a correct knowledge of it, that its reporters should have precisely stated the motive condition of the vascular system during the disease, whether the pulse had been much accelerated, invigorated, or enfeebled. If the arterial action should have been much excited, (as is not unusual in gouty affection) was it strictly compatable with the refrigerant treatment locally adopted, to stimulate the system by cordial medicine? The sensations of acute pain in the head and stomach, may be justly supposed to have irritated the powers of life too much of themselves, without the farther aid of medicinal excitement. It is not my wish to suggest the slightest disapprobation to a practice which appears to have been directed by *exemplary candour* and *humanity*, but merely to point out the propriety of combating all occurring symptoms, according to their seeming nature. Thus, if violent pain were to occur in either the head, stomach, or any other vital organ, during the refrigerant treatment of gout, it surely would not be strictly consistent with the intention of cure to aggravate the excessive irritation by additional excitement. On the contrary, if a faultering weak pulse should present with spasmodic twitches fleeting through the frame, and either a general or partial sense of deficient heat, it would be advisable to sustain, by the judicious use of stimulant agents, the declining energies of life, until the salutary balance of motive power might be reinstated.

The reporters of Mr. Mace's case will hereafter have to direct their best judgment to the most effectual means of destroying the morbid association, which seems to be established between the inflammatory affection of the joints and the irritation of the viscera. In doing this, they will indeed have to cope with one of the principal evils of prolonged gout, but will probably have nothing to risk if they guide their practice by both the local and general indication of prevailing temperature. The prompt removal of pain would be also preferable to its gradual decline, which may be considered as rather an actual though imperceptible extension of it to such parts as happen to be most susceptible of morbid excitement, than its complete annihilation.

The refrigerant treatment of gout is now conducted by either sudden immersion or affusion of the affected parts in cold water, unremittedly continued, until all sense of painful heat be subdued. This decisive mode of practice expedites the salutary efficacy of the treatment, by at once extinguishing the inflammatory heat, and preventing its noxiously stimulant effects being propagated to the system. Instances are now in my possession, and soon will be published, of patients keeping the affected parts in a deep vessel of water for upwards of an hour at a time, and recurring to it with equal duration on the least return of pain. In this way effectual relief has been obtained without in the slightest manner affecting the general health.

It should not for a moment be forgotten, that gout is a disease of excessive temperature, that it is of local origin, and that its sympathetic influence on the system should be likewise treated according to the existing circumstances of heat or excitement; if redundant, it should be diminished; if deficient, supplied. Gout thus managed may, with all its *anomalies*, be confidently encountered; and will be found, even in its most inveterate and difficult forms, safely and efficaciously remediable by the refrigerant mode of treatment.

Your correspondent Mr. Mantal has, in his remarks on Mr. Edlin's account of Mr. Baker's case in your last Journal, exhibited an *unprejudiced* and an *enlightened* view of the nature of gout. He has very judiciously explained the way in which motive irregularity may morbidly affect the healthful conditions of life, and has (as more largely illustrated in my Dissertation on Gout) evinced the propriety of treating gouty inflammation, in both its original attack and sympathetic extension, as a disease of temperature, withholding and adding excitement according to the prevailing sensations of either heat or cold.

My opinion of the nature and cure of gout does not essentially differ from that of your ingenious Correspondent; his intelligent observations therefore on the subject, cannot fail further to elucidate the cooling treatment of that disease.

The want of liberality in Mr. Edlin's statement of Mr. Baker's case, unwillingly compelled me to suspect the purity of his motive in ushering it into public notice, to dispute its credibility, and to offer objections to the conclusion attempted to be deduced from it.

The

The umpirage of the controversy now rests with the Public. Mr. Edlin's Case and my Reply to it are both submitted to that high arbitration, the decision of which, and the accumulating evidence of farther experience, must sooner or later irrefragably determine the truth of the question. I am, &c.

*Taunton, Feb.* 12, 1805. ROBERT KINGLAKE.

